# Socioeconomic status is associated with symptom severity and sickness absence in people with infectious intestinal disease in the UK

**DOI:** 10.1186/s12879-017-2551-1

**Published:** 2017-06-23

**Authors:** Tanith C. Rose, Natalie L. Adams, Benjamin Barr, Jeremy Hawker, Sarah J. O’Brien, Mara Violato, Margaret Whitehead, David C. Taylor-Robinson

**Affiliations:** 1 NIHR Health Protection Research Unit in Gastrointestinal Infections, Liverpool, UK; 2 Department of Public Health and Policy, University of Liverpool, Liverpool, UK; 3 National Infection Service, Public Health England, London/Birmingham, UK; 4 Health Economics Research Centre, University of Oxford, Oxford, UK; 5 Department of Public Health and Policy Institute of Psychology, Health and Society, University of Liverpool, Whelan Building, Liverpool, L69 3GB UK

**Keywords:** Socioeconomic factors, Occupation, Infectious intestinal disease, Diarrhoea, Sick leave, Symptom severity

## Abstract

**Background:**

The burden of infectious intestinal disease (IID) in the UK is substantial. Negative consequences including sickness absence are common, but little is known about the social patterning of these outcomes, or the extent to which they relate to disease severity.

**Methods:**

We performed a cross-sectional analysis using IID cases identified from a large population-based survey, to explore the association between socioeconomic status (SES) and symptom severity and sickness absence; and to assess the role of symptom severity on the relationship between SES and absence. Regression modelling was used to investigate these associations, whilst controlling for potential confounders such as age, sex and ethnicity.

**Results:**

Among 1164 cases, those of lower SES versus high had twice the odds of experiencing severe symptoms (OR 2.2, 95%CI;1.66–2.87). Lower SES was associated with higher odds of sickness absence (OR 1.8, 95%CI;1.26–2.69), however this association was attenuated after adjusting for symptom severity (OR 1.4, 95%CI;0.92–2.07).

**Conclusions:**

In a large sample of IID cases, those of low SES versus high were more likely to report severe symptoms, and sickness absence; with greater severity largely explaining the higher absence. Public health interventions are needed to address the unequal consequences of IID identified.

**Electronic supplementary material:**

The online version of this article (doi:10.1186/s12879-017-2551-1) contains supplementary material, which is available to authorized users.

## Background

Infectious intestinal disease (IID) is extremely common, with an estimated 17 million sporadic cases occurring each year in the United Kingdom (UK) [1]. It also confers significant morbidity and associated healthcare costs. Around half of those who experience IID report absence from work or school which amounts to an estimated loss of nearly 19 million days per annum, with potential ramifications for adult earnings and child education [2]. Additionally, there are approximately one million general practice (GP) consultations for IID every year in the UK [1]. The burden of IID is clearly evident, yet relatively little is known about the extent of socioeconomic inequalities in the clinical, social and economic consequences of IID.

Studies conducted in developed countries, suggest individuals of low socioeconomic status (SES) compared to high, have higher rates of GP consultation [3, 4] and hospital admission due to IID [5–9]. For example, in the West Midlands in the UK, hospital admission rates for young children with IID were twice as high in the most deprived areas compared to the least [6]. However, the mechanisms explaining these apparent health inequalities are unknown. Contributing factors may include differential risk of infection, healthcare seeking behaviour, or disease severity across socioeconomic groups.

Separating out the effects of these potential explanations is imperative to understand the role they play in generating the inequalities observed, and so that interventions and policies can be developed to tackle the problem. A cross-sectional analysis of IID cases identified in the English IID1 studies, showed that IID cases of lower SES (as measured by educational attainment) were more likely to present to their GP for an episode of IID, compared to those of higher SES [3]. In addition, disease severity was strongly predictive of GP presentation for IID, however numbers were insufficient to assess the relationship between SES and IID severity. These findings indicate that healthcare seeking behaviour for IID may be socially patterned, which potentially could be related to disease severity.

Negative consequences of IID also include sickness absence, which may or may not be related to IID severity. Rates of general (all cause) sickness absence, have been shown to be higher for those of lower SES compared to high [10], however some studies have demonstrated that this association can in part be explained by the increased levels of morbidity for those of lower SES [10, 11]. The few studies that have investigated the relationship between SES and sickness absence due to IID have produced conflicting results [12, 13]; and we are yet to find a study that has examined the role of IID severity on the relationship between SES and sickness absence. To gain a better understanding of inequalities in the consequences of IID, we analysed a large sample of IID cases to explore the association between SES and measures of self-reported IID symptom severity and sickness absence.

## Methods

### Study design and data source

We analysed cases of IID identified in the population-based IID2 study. The IID2 study was conducted across the UK in 2008–9 and contained several studies, the methods of which have been described in detail elsewhere [14]. The IID2 study was granted ethical approval by the North West Research Ethics Committee (07/MRE08/5) [15]. Participants gave written informed consent for their anonymised data to be used for future analyses.

The IID2 study contained two major components; a prospective cohort study and a GP presentation study. For the cohort study, patients were randomly selected from the registers of 88 general practices and invited to participate. Participants completed a baseline questionnaire containing questions on socio-demographic factors, and were followed-up weekly for one year to determine the incidence of IID. Incident cases completed symptom questionnaires including questions on symptom severity, absenteeism and recent foreign travel. For the GP presentation study, all patients who consulted their GP for an episode of IID across 37 of the 88 general practices, over a one year period were invited to participate in a survey which included the same socio-demographic and symptom questions as the former study.

Cases identified via both components of the IID2 study were combined for this analysis. Cases of IID were defined as people aged five years or older, with loose stools or clinically significant vomiting lasting less than two weeks, in the absence of a known non-infectious cause, preceded by a symptom-free period of three weeks [14]. We included cases aged five years or older, to limit potential misclassification of the more subjective symptoms, such as headache and nausea, in young children (see below details of symptom severity score). For cases meeting the case definition, all recurrent episodes of IID were removed regardless of the timeframe between episodes. If a case experienced more than one episode of IID during follow-up, only information related to the first episode was retained to create a sample of independent observations.

### Outcomes and covariates

The outcomes of interest were symptom severity and sickness absence due to IID. The symptom severity score was derived from information on the presence/absence of nine symptoms, and the duration of four symptoms, which were self-reported by the cases, using previously published methods [3]. In brief, the presence and duration scores were multiplied, and the resulting product scores summed across the symptoms, creating an overall symptom severity score for each case (Additional file 1). The symptom severity variable was converted into tertiles, whereby three approximately equally sized groups were created according to the distribution of the severity score [3]. The second outcome of interest was sickness absence; a binary variable indicating whether the episode of IID prevented the case from going to work or school. Sickness absence was only defined for cases of school or working age (aged five years or older, and up to 60 years for women and 65 years for men, as older age groups were unlikely to be in work or education [16]).

The main exposure of interest was SES measured at the individual-level using the National Statistics Socioeconomic Classification (NS-SEC) [17]. The NS-SEC was designed to take into account the nature of modern inequalities, by measuring conditions of occupations and also employment relations [17, 18]. To derive the NS-SEC, participants answered via self-completion questionnaire, questions relating to the occupation and employment status of the main-earner in their household, reporting on the main-earner’s current or last main job. Individuals were assigned the category ‘Not classifiable’ if information was missing and as such an NS-SEC class could not be calculated. We re-coded the five-class NS-SEC version to form the three-class version which can be assumed to have a hierarchy [17]. The classes from high to low SES represented managerial/professional, intermediate and routine/manual occupations.

Potential confounding variables of the relationship between SES, symptom severity and sickness absence included in the analysis, were age, sex, ethnicity, foreign travel in the ten days before disease onset, and urban/rural residency (based on Super Output Areas) [19].

### Statistical analysis

Ordinal logistic regression was employed for the symptom severity outcome, and logistic regression for the binary absence outcome. Model parameters were estimated by maximum likelihood. For the ordinal logistic regression models, the proportional odds assumption was assessed using graphical methods [20, 21]. Generalised additive models (GAMs) were used to assess the linear relationship between the continuous age variable and the outcomes (Additional file 1). There was a linear relationship between age and the log-odds of sickness absence, therefore age was included as a continuous variable when modelling the absence outcome. The relationship between age and symptom severity was non-linear, therefore a categorical age group variable was included when modelling symptom severity.

A hierarchical approach was used for the multivariate regression modelling. Firstly, we fitted baseline models for each of our two outcomes (symptom severity and sickness absence) with age, sex and ethnicity as independent variables. Secondly, we added NS-SEC as an additional independent variable to the models and tested the improvement in model fit using generalised likelihood ratio statistics to compare nested models. Thirdly, we tested whether the inclusion of additional confounders (recent foreign travel and urban/rural residency) improved the model fit. Finally, to explore whether differences in disease severity explained any association between NS-SEC and sickness absence, we added symptom severity as a control variable to the model with sickness absence as an outcome.

Listwise deletion was used as the method of handling missing data. For the two outcomes, cases with missing data within any of the variables to be included in the models were excluded. Sensitivity analyses were performed using multiple imputation by chained equations to impute missing data values for all of the variables included in the models.

We undertook several robustness tests, repeating our analyses using alternative cut-offs for the symptom severity categories; including recurrent episodes of IID within the same individual; using cases of all ages; and stratifying results by child and adult age groups. We also examined the appropriateness of combining cases from the IID2 cohort and GP presentation studies. Analyses were conducted using R (version 3.3.1).

## Results

The IID2 studies identified 1915 cases meeting our inclusion criteria of which 1270 were of school or working age and included in the sickness absence analysis (see Additional file 1 for flow diagram). Characteristics of the cases stratified by NS-SEC are shown in Table 1. Around half of cases were in managerial/professional occupations, and the vast majority were of White ethnicity (>90%). Cases in routine/manual compared to managerial/professional occupations were less likely to reside in rural areas, be female or have travelled abroad before their illness. Age and ethnicity were not associated with NS-SEC.

**Table 1:** Unadjusted distribution of each variable by NS-SEC category, for the two analysis samples (IID2 study 2008–9)

	Cases ≥5 years of age (*n* = 1915)					Cases school/working age (*n* = 1270)				
	Percentage within each category of NS-SEC					Percentage within each category of NS-SEC				
	Managerial/ professional	Intermediate	Routine/manual	*p*-value^a^	All cases^b^	Managerial/professional	Intermediate	Routine/ manual	*p*-value^a^	All cases^b^
	(*n* = 949)	(*n* = 330)	(*n* = 337)		(*n* = 1915)	(*n* = 662)	(*n* = 215)	(*n* = 228)		(*n* = 1270)
Age group (years)										
5–14	12.2	9.4	9.5	0.342	10.6	17.5	14.4	14	0.337	16
15–24	4.4	5.2	7.4		5.2	6.3	7.9	11		7.9
25–44	24.1	22.7	23.7		22.9	34.6	34.9	35.1		34.5
45–64	36	36.7	33.5		35.1	41.5	42.8	39.9		41.7
65+	23.2	26.1	25.8		26.2					
Male	38	32.7	45.1	0.004	37.9	39.7	37.2	49.6	0.014	41
Ethnicity Non-White	3.4	4.8	3.6	0.468	4	3.9	7	4.8	0.185	5.1
Rural residence	30.6	30	19	<0.001	28.8	30.3	28.4	20.6	0.019	27.6
Travelled before illness	14.3	10.3	7.7	0.004	11.9	15.6	13.1	7.9	0.013	12.9
Symptom severity										
Mild	38.3	34.4	20.3	<0.001	24.5	40	36	20.7	<0.001	26.8
Moderate	34	33	35.5		24.9	36.7	32	38		27.1
Severe	27.7	32.6	44.2		23.4	23.3	32	41.3		22.1
Absent work/school	55.1	54.2	63	0.028	53.4	61.4	62.7	71.6	0.023	61

*IID* infectious intestinal disease, *NS-SEC* National Statistics Socioeconomic Classification, *SD* standard deviation

Figures expressed as percentages except where stated otherwise

^a^Statistical significance of relationship between NS-SEC and each variable, tested using χ^2^ test

^b^Total number of cases includes those with missing NS-SEC

Missing data (%) for cases ≥5 years and cases school/working age, respectively: NS-SEC 16 and 13; Urban/rural 0.1 and 0.2; Foreign travel 0.6 and 0.3; Symptom severity 27 and 24; Absence 3.6 and 2.7

### Symptom severity

The symptom severity score ranged from 2 to 40 and was positively skewed. The boundaries for the tertiles were: mild (score 2–9), moderate (score 10–15) and severe (score 16–40). In total, 1164 (61%) cases had complete data for the variables of interest.

The univariate associations between symptom severity and the exposures are shown in Table 2, and two nested multivariate models for symptom severity are displayed in Table 3. The addition of NS-SEC to the baseline model improved the model fit when comparing the likelihoods of the models (Likelihood ratio χ^2^ 31.7; *P* < 0.001). For those in routine/manual compared to managerial/professional occupations the odds of experiencing severe IID symptoms, versus mild or moderate symptoms combined, were two times greater (OR 2.2, 95%CI;1.66–2.87). The odds of experiencing severe symptoms were greater for those of Non-White compared to White ethnicity, and for those aged 15–24 years compared to 5–14 years, however these estimates were based on small numbers (43 cases were of Non-White ethnicity; 61 cases were aged 15–24 years). There was no improvement in the model fit when the variables urban/rural residency and recent foreign travel were added to the Baseline + NS-SEC model, and therefore these models are not presented.

**Table 2:** Univariate associations for IID symptom severity and sickness absence outcomes (IID2 study 2008–9)

	Severe symptoms versus mild or moderate symptoms combined OR (95%CI)	Sickness absence versus no sickness absence OR (95%CI)^a^
	Cases with complete data ≥5 years of age (*n* = 1164)	Cases with complete data school/working age (*n* = 818)
Age group (years)		
5–14	reference	
15–24	2.88 (1.59–5.31)	
25–44	0.99 (0.68–1.45)	
45–64	0.70 (0.49–1.01)	
65+	0.60 (0.41–0.89)	
Age (years)		0.98 (0.97–0.99)
Sex		
Female	reference	reference
Male	0.92 (0.74–1.14)	0.94 (0.70–1.25)
Ethnicity		
White	reference	reference
Non-White	2.27 (1.28–4.10)	3.13 (1.38–8.41)
NS-SEC		
Managerial/professional	reference	reference
Intermediate	1.21 (0.92–1.61)	1.13 (0.78–1.66)
Routine/manual	2.18 (1.67–2.86)	1.77 (1.22–2.58)
Residence		
Urban	reference	reference
Rural	0.82 (0.65–1.03)	0.98 (0.71–1.34)
Travelled before illness		
No	reference	reference
Yes	1.21 (0.88–1.66)	0.66 (0.44–0.99)
Symptom severity		
Mild		reference
Moderate		3.88 (2.75–5.51)
Severe		5.99 (4.07–8.95)

*CI* confidence interval, *IID* infectious intestinal disease, *NS-SEC* National Statistics Socioeconomic Classification, *OR* odds ratio

^a^Since the absence outcome was common, the odds ratios should not be interpreted as risk ratios

**Table 3:** Multivariate models for severe IID symptoms, versus mild or moderate symptoms combined for cases ≥5 years of age (IID2 study 2008–9)

	Baseline model	Baseline + NS-SEC
	OR (95%CI)	OR (95%CI)
Age group (years)		
5–14	reference	reference
15–24	3.01 (1.66–5.57)	2.70 (1.48–5.02)
25–44	1.02 (0.69–1.50)	0.96 (0.65–1.42)
45–64	0.73 (0.51–1.06)	0.69 (0.47–1.00)
65+	0.64 (0.43–0.95)	0.60 (0.41–0.90)
Sex		
Female	reference	reference
Male	0.95 (0.77–1.19)	0.90 (0.72–1.13)
Ethnicity		
White	reference	reference
Non-White	2.11 (1.18–3.83)	2.03 (1.14–3.70)
NS-SEC		
Managerial/professional		reference
Intermediate		1.21 (0.91–1.61)
Routine/manual		2.18 (1.66–2.87)
Log-likelihood	−1255.1	−1239.3
Deviance	2510.2	2478.6
AIC	2526.2	2498.6
BIC	2566.7	2549.2
Number	1164	1164

*AIC* Akaike information criterion, *BIC* Bayesian information criterion, *CI* confidence interval, *IID* infectious intestinal disease, *NS-SEC* National Statistics Socioeconomic Classification, *OR* odds ratio

### Sickness absence

Of the 1270 cases of school or working age, 818 (64%) had complete data for the variables of interest (Additional file 1). Over half of the cases (62%) were absent from work or school following their illness. Amongst the absentees, the majority took 1–2 days sick leave (62%), and few took more than five days (8%).

The univariate associations between sickness absence and the exposures are shown in Table 2, and three nested multivariate models for sickness absence are displayed in Table 4. The addition of NS-SEC produced a better fitting model compared to the baseline model (Likelihood ratio χ^2^ 10.2; *P* = 0.006). Those in routine/manual compared to managerial/professional occupations had a higher odds of absence (OR 1.8, 95%CI;1.26–2.69).

**Table 4:** Multivariate models for sickness absence due to IID for cases of school/working age (IID2 study 2008–9)

	Baseline model	Baseline + NS-SEC	Baseline + NS-SEC + Severity
	OR (95%CI)^a^	OR (95%CI)^a^	OR (95%CI)^a^
Age (years)	0.98 (0.98–0.99)	0.98 (0.98–0.99)	0.99 (0.98–1.00)
Sex			
Female	reference	reference	reference
Male	0.95 (0.71–1.28)	0.91 (0.68–1.22)	0.92 (0.67–1.26)
Ethnicity			
White	reference	reference	reference
Non-White	2.66 (1.16–7.22)	2.58 (1.12–7.00)	1.91 (0.80–5.31)
NS-SEC			
Managerial/professional		reference	reference
Intermediate		1.13 (0.77–1.66)	1.05 (0.70–1.59)
Routine/manual		1.83 (1.26–2.69)	1.38 (0.92–2.07)
Symptom severity			
Mild			reference
Moderate			3.60 (2.54–5.14)
Severe			5.27 (3.54–7.93)
Log-likelihood	−531.0	−525.9	−482.4
Deviance	1062.0	1051.9	964.9
AIC	1070.0	1063.9	980.9
BIC	1088.9	1092.1	1018.5
Number	818	818	818

*AIC* Akaike information criterion, *BIC* Bayesian information criterion, *CI* confidence interval, *IID* infectious intestinal disease, *NS-SEC* National Statistics Socioeconomic Classification, *OR* odds ratio

^a^Since the absence outcome was common, the odds ratios should not be interpreted as risk ratios

When symptom severity was added to this model the odds of absence for those in routine/manual compared to managerial/professional occupations was attenuated and rendered non-significant (OR 1.4, 95%CI;0.92–2.07). There was a dose-response relationship between symptom severity and the odds of absence. Those with severe compared to mild symptoms had five times the odds of absence (OR 5.3, 95%CI;3.54–7.93). Again, there was no improvement in the model fit when the variables urban/rural residency and recent foreign travel were added to the Baseline + NS-SEC model, and therefore these models are not presented.

### Sensitivity analyses

We undertook several robustness tests. Similar results to those reported were observed when analyses were conducted with recurrent episodes of IID included with clustering at the individual level accounted for using mixed-effects models, and when the boundaries of the symptom severity categories were changed so that there was an equal 12 point severity score difference within each category (data not shown). Results from multiply imputed datasets, and analyses involving cases of all ages and stratified results by child and adult age groups, also confirmed those from the main analyses (Additional file 1), however ethnicity was not associated with symptom severity when analyses were performed using the imputed datasets. Additionally, comparable associations were found when investigating predictive factors for the duration of absence among absentees (Additional file 1). Lastly, the appropriateness of combining cases from the IID2 component studies was supported by analyses indicating the relationships between NS-SEC and the outcomes were not significantly different between the cohort and GP presentation studies (Additional file 1).

## Discussion

We analysed data from the largest population-based survey of IID conducted in the UK, and found that IID cases of lower SES compared to high were more likely to experience severe symptoms, and were more likely to be absent from work or school. The association between SES and sickness absence was largely explained by greater symptom severity amongst the more disadvantaged groups.

Our findings are comparable to those of other studies that have analysed measures of IID severity and SES, however these studies are sparse in number, and have tended to focus on children under five years of age. Our findings suggest that the association between SES and IID severity is true for the whole (all age) population, not just for young children. We identified one British study which analysed data from the population-based Avon Longitudinal Study of Pregnancy and Childhood (ALSPAC), to assess predictive factors for the duration of diarrhoeal episodes in children less than six months of age [22]. The authors found that infants living in rented versus mortgaged/owned accommodation (a suggested indicator of SES) had greater odds of experiencing diarrhoea for six or more days. However, this association became non-significant after adjustment for duration of breast feeding, with longer spells of breast feeding providing protection against prolonged diarrhoea.

Whilst very few cases were admitted to hospital in our sample (<1%), our findings are somewhat similar to those of studies conducted in hospital settings. At this severe end of the disease spectrum, one UK-based study found low SES was associated with longer time to discharge for children hospitalised with gastroenteritis in univariate analysis [23]. Similarly, among American children less than five years of age hospitalised with gastroenteritis, those enrolled in Medicaid (a proxy measure for low SES) experienced longer average length of stay, compared to children not enrolled, when no other factors were taken into consideration [24]. In contrast, multivariate analysis revealed that education level and income were not related to length of stay for Canadian children less than five years of age hospitalised with rotavirus gastroenteritis, whereas regularly seeing a physician for a medical condition was associated with longer hospital stays [25].

These findings might suggest that the association between SES and IID severity could be mediated by socially patterned factors that impair immune response, such as lack of breast feeding in infancy and multimorbidity [26, 27], both of which are more prevalent among lower socioeconomic groups [28, 29]. Additional biologically plausible mechanisms which might help to explain a greater burden of severe IID in lower socioeconomic groups, but are as yet to be substantiated in this context, include increased levels of chronic stress, smoking, and nutritional deficiencies, all of which display social gradients and are associated with immune system compromise [30–34]. The potential mediating role of immune suppressing variables on the relationship between SES and symptom severity warrants further investigation.

We found IID cases of lower SES compared to high had greater odds of sickness absence due to IID, and this was largely explained by greater symptom severity amongst cases of lower SES. In a cohort of UK civil servants, age adjusted rates of sickness absence due to gastroenteritis, were over six and four times higher for men and women respectively, in lower employment grades compared to high [12]. Conversely, self-reported sickness absence for gastroenteritis in a cohort of Dutch employees was unrelated to education level in univariate analysis [13]. These conflicting findings may, in part, be due to the different populations studied, since our age, sex and ethnicity adjusted results for absence were akin to those observed in the UK-based study of civil servants [12]. However, neither study investigated the role of symptom severity, which was identified as an important mediator of the relationship between SES and sickness absence in our analysis.

There are several limitations to this analysis. The validity of our results depended upon the unbiased and accurate self-reporting of symptoms and sickness absence among cases. If those of lower SES perceived their symptoms differently to those of higher SES, which has been observed in studies investigating perceptions of pain across socioeconomic groups [35, 36], our results could be a mere artefact of the severity measurement. Nonetheless, the variables used to derive the symptom severity score in our study were related to the presence and duration of symptoms, which are rather more objective measures of severity compared to, for example, a subjective rating of symptom severity from mild to severe.

There was a large amount of missing data, particularly within the NS-SEC and symptom severity variables (Table 1). Listwise deletion as a method of handling missing data can produce unbiased estimates when data are missing completely at random [37]. However, the odds of whether data were missing or not within the NS-SEC and symptom severity variables, were associated with other variables within the dataset, supporting the idea that missing data were missing at random, rather than missing completely at random. Sensitivity analyses were therefore performed using multiple imputation by chained equations to impute missing data values (Additional file 1). Results from multiply imputed datasets confirmed those from the main analyses, suggesting that any bias resulting from the use of listwise deletion, was minimal. Ethnicity however was not associated with symptom severity when analyses were performed using the imputed datasets.

Cases identified in the IID2 cohort and GP presentation studies were combined for this analysis. Individuals in managerial/professional occupations, those aged 55+ years and those of White ethnicity were over-represented in the cohort study compared to the UK population, and individuals in intermediate and routine/manual occupations and those aged 15–24 years in particular were under-represented [15]. Under-representation of lower socioeconomic groups is commonplace in population-based surveys [38], and could limit the external validity of our findings. Nevertheless, the internal validity of our findings should remain unaffected. It is possible that if non-participation or the design of the studies resulted in the under-representation of cases of lower SES who experienced milder symptoms, we may have overestimated the association between low SES and severe symptoms. However, within the cohort study this is unlikely as cases were captured prospectively. The GP presentation study may have been more prone to selection bias, since cases with more severe symptoms and those of lower SES may be more likely to present to their GP for an episode of IID [3], however as shown in Additional file 1, the relationship between NS-SEC and symptom severity was not significantly different between the cohort and GP presentation studies.

There is the potential for different pathogens to infect people of different SES, for example *Listeria* and norovirus have been associated with low SES in some studies [39, 40]. Unfortunately, we were unable to explore the role of pathogen type on the association between SES and symptom severity because for around 58% of the sampled cases no pathogen was identified [15]. The impact of pathogen type on the association between SES and symptom severity is unknown, however the severity of illness likely depends not only on the infecting pathogen but also on host factors and the dose to which the host is exposed [41]. The relationship between SES, pathogen type and IID symptom severity could be explored using a larger sample of cases, since for the majority a pathogen will not be identified.

Finally, the IID2 study also contained a retrospective telephone survey which gave higher IID incidence estimates compared to the IID2 cohort study [15], however we were unable to repeat our analyses with cases identified in the telephone survey because NS-SEC information was not collected. We were also unable to assess inequalities in sickness absence amongst those providing care for IID cases (caregiver informative was not collected) however this may be an interesting avenue for further research.

## Conclusions

Our study sheds new light into an under-researched area and indicates that the consequences of having an IID may be unequally shared across socioeconomic groups. These consequences are potentially serious. Loss of working days due to sickness can have important economic consequences and these are likely to be more severe for more disadvantaged groups who might receive less adequate compensation from their employer. Loss of days from school can affect educational attainment [42], suggesting that the unequal effects of IID could exacerbate educational inequalities. Actions that reduce the risk of acquiring IID are unlikely to sufficiently address these inequalities; public health interventions also need to reduce their unequal consequences. Further research is required to understand the mechanisms explaining greater severity of illness in disadvantaged groups, and to identify ways to minimise the differential impact of IID on sickness absence.

## Additional file

Additional file 1: Supplementary material and sensitivity analyses. (PDF 387 kb)

## Abbreviations

ALSPAC: Avon Longitudinal Study of Pregnancy and Childhood
GAM: Generalised additive model
GP: General practice
IID: Infectious intestinal disease
NS-SEC: National Statistics Socioeconomic Classification
SES: Socioeconomic status
UK: United Kingdom
