# Supplementary material for: Socioeconomic status is associated with symptom severity and sickness absence in people with infectious intestinal disease in the UK
Source: BMC Infect Dis. 2017 Jun 23;17:447. doi: 10.1186/s12879-017-2551-1 (PMC5481911; doi:10.1186/s12879-017-2551-1)
Supplement: Supplementary file 1 — Supplementary material and sensitivity analyses. (PDF 387 kb) [file 12879_2017_2551_MOESM1_ESM.pdf]

Supplementary Material

Appropriateness of Combining IID Cases from the Component Studies of the IID2 Study

To evaluate whether the relationships between NS-SEC and the outcomes differed between the cohort and GP presentation studies of the IID2 study, an interaction term was added to the most parsimonious multivariate models for the IID symptom severity and sickness absence outcomes identified in the main analysis. This interaction term between NS-SEC (the primary exposure of interest) and study type, was not statistically significant in the models, indicating that the relationships between NS-SEC and the outcomes were not significantly different between the cohort and GP presentation studies.

Multivariate model for severe IID symptoms, versus mild or moderate symptoms combined for cases ≥5 years of age, with interaction term between NS-SEC and study type (IID2 study 2008–9)

	**Odds ratio (95% CI)**	**p-value**
**Age group** 15–24 years [a]	1.49 (0.79–2.87)	0.22166
**Age group** 25–44 years [a]	0.70 (0.46–1.06)	0.09449
**Age group** 45–64 years [a]	0.66 (0.44–0.98)	0.04078
**Age group** 65+ years [a]	0.42 (0.27–0.65)	<0.0001
**Sex** Male [b]	0.60 (0.47–0.76)	<0.0001
**Ethnicity** Non-White [c]	1.23 (0.66–2.33)	0.51992
**NS-SEC** Intermediate [d]	0.81 (0.54–1.21)	0.30585
**NS-SEC** Routine/manual [d]	1.39 (0.97–2.01)	0.07591
**Study type** Cohort [e]	0.08 (0.06–0.12)	<0.0001
**Interaction term** (NS-SEC Intermediate:Cohort)	**1.23 (0.66–2.25)†**	**0.51123**
**Interaction term** (NS-SEC Routine/manual:Cohort)	**0.94 (0.50–1.73)†**	**0.83392**

Number: 1164 IID cases included in model
CI = confidence interval; IID = infectious intestinal disease; NS-SEC = National Statistics Socioeconomic Classification
[a] reference category = Age group 5–14 years
[b] reference category = Sex Female
[c] reference category = Ethnicity White
[d] reference category = NS-SEC Managerial/professional occupations
[e] reference category = Study type GP presentation
† Ratio of odds ratios

Multivariate models for sickness absence due to IID for cases of school/working age, with interaction term between NS-SEC and study type (IID2 study 2008–9)

	**Odds ratio (95% CI)**	**p-value**
**Age** (years)	0.99 (0.98–1.00)	0.01267
**Sex** Male [a]	0.86 (0.63–1.19)	0.37066
**Ethnicity** Non-White [b]	1.76 (0.74–4.90)	0.23599
**NS-SEC** Intermediate [c]	0.91 (0.51–1.65)	0.75240
**NS-SEC** Routine/manual [c]	1.26 (0.75–2.16)	0.38271
**Symptom severity** Moderate [d]	3.13 (2.16–4.57)	<0.0001
**Symptom severity** Severe [d]	4.02 (2.49–6.54)	<0.0001
**Study type** Cohort [e]	0.65 (0.41–1.02)	0.05991
**Interaction term** (NS-SEC Intermediate:Cohort)	**1.24 (0.55–2.80)†**	**0.60242**
**Interaction term** (NS-SEC Routine/manual:Cohort)	**1.03 (0.44–2.40)†**	**0.94993**

Number: 818 IID cases included in model
CI = confidence interval; IID = infectious intestinal disease; NS-SEC = National Statistics Socioeconomic Classification
[a] reference category = Sex Female
[b] reference category = Ethnicity White
[c] reference category = NS-SEC Managerial/professional occupations
[d] reference category = Symptom severity Mild
[e] reference category = Study type GP presentation
† Ratio of odds ratios

Symptom Severity Scoring System

The symptom severity score was derived from information on the presence/absence of nine symptoms, and the duration of four symptoms, which were self-reported by the cases. The presence and duration scores were multiplied, and the resulting product scores summed across the symptoms, creating an overall symptom severity score for each case.

Symptoms that were used to derive the symptom severity score

	**Present**		**Duration in days**			
**Symptoms**	Yes	No	1–2	3–4	5–6	7+
Diarrhoea	2	0	1	2	3	4
Vomiting	2	0	1	2	3	4
Diarrhoea with blood	3	0	1	2	3	4
Nausea	2	0	1	2	3	4
Abdominal cramps†	3	0	-	-	-	-
Loss of appetite†	2	0	-	-	-	-
Fever (high temperature)†	3	0	-	-	-	-
Cough or runny/blocked nose or sore throat†	1	0	-	-	-	-
Headache†	2	0	-	-	-	-

† Duration data unavailable, a duration score of 1 was applied when calculating the severity score

Generalised Additive Models

Generalised additive models showing the univariate relationships between age and the IID symptom severity and sickness absence outcomes

Y axis in the plots shows scaled log-odds of: a). Symptom severity and b). Sickness absence

a). Symptom severity

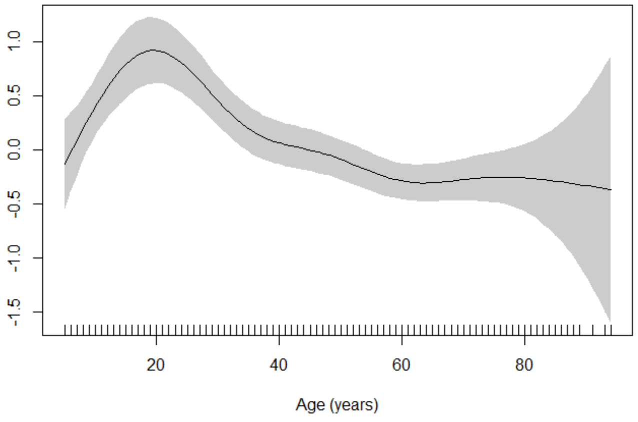

b). Sickness absence

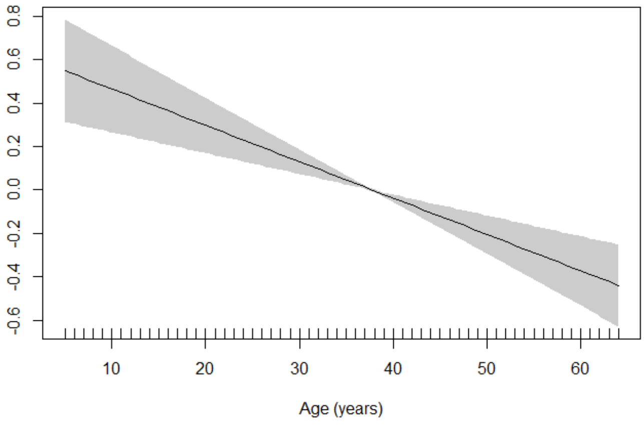

Flow Diagram of Participants Included in the Analyses

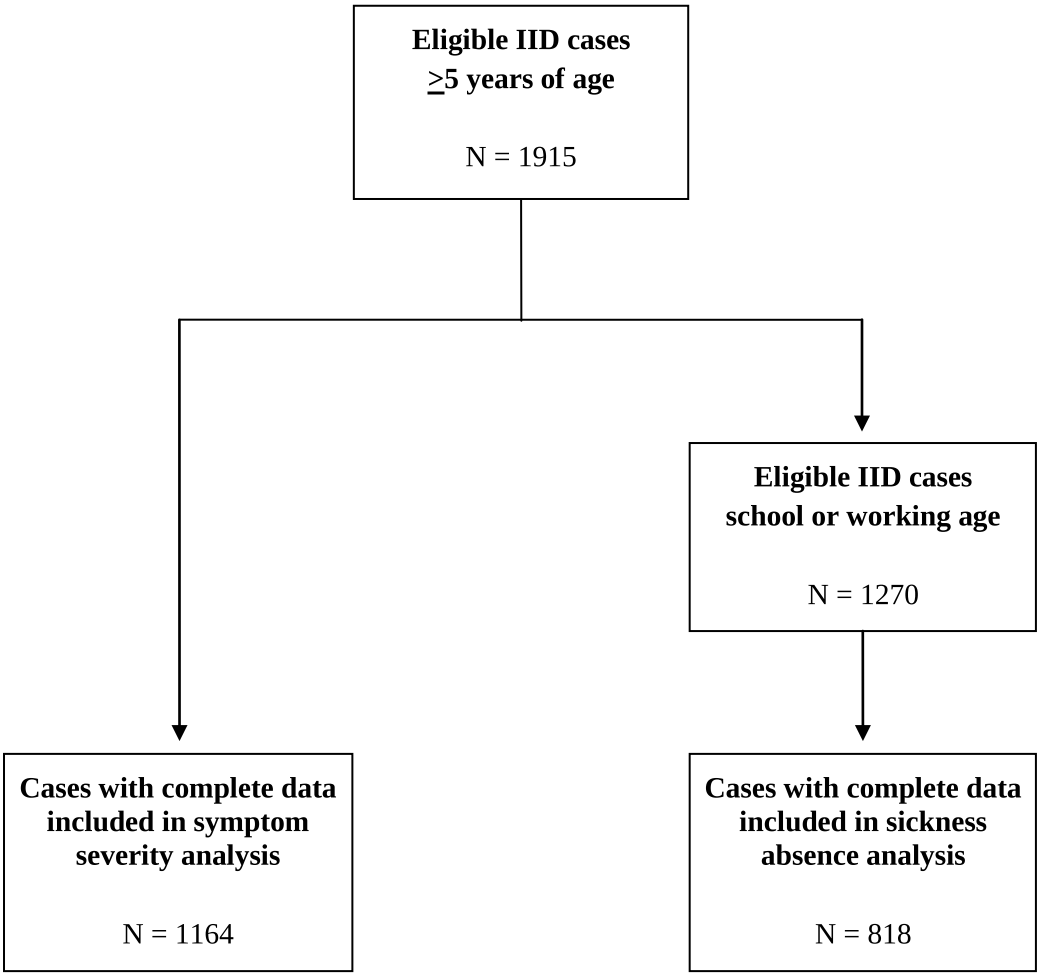

Sensitivity Analysis with Cases of All Ages and Stratified Results by Age

Multivariate analyses using the most parsimonious models for the IID symptom severity and sickness absence outcomes identified in the main analysis were repeated without applying age restrictions to the sample. Results stratified by children and adult age groups with the same lower and upper age limits as utilised in the main analysis are also presented.

Multivariate models for severe IID symptoms, versus mild or moderate symptoms combined for cases of all ages and stratified by child and adult age groups (IID2 study 2008–9)

	**All ages**<br>OR (95%CI)	**Children**<br>**≥5 to <16 years**<br>OR (95%CI)	**Adults**<br>**≥16 years**<br>OR (95%CI)
**Age (years)**	0.99 (0.99–1.00)	1.16 (1.02–1.31)	0.98 (0.97–0.99)
**Sex**			
Female	reference	reference	reference
Male	0.88 (0.72–1.09)	1.13 (0.58–2.20)	0.91 (0.72–1.15)
**Ethnicity**			
White	reference	reference	reference
Non-White	1.76 (1.04–2.99)	3.26 (1.04–10.77)	1.34 (0.68–2.68)
**NS-SEC**			
Managerial/professional	reference	reference	reference
Intermediate	1.22 (0.93–1.59)	0.82 (0.31–2.06)	1.24 (0.92–1.67)
Routine/manual	2.13 (1.65–2.75)	2.96 (1.22–7.48)	2.12 (1.60–2.83)
Number	1264	129	1035

CI = confidence interval; IID = infectious intestinal disease; NS-SEC = National Statistics Socioeconomic Classification; OR = odds ratio

Multivariate models for sickness absence due to IID for cases of all ages and stratified by child and adult age groups (IID2 study 2008–9)

	**All ages**<br>OR (95%CI)	**Children**<br>**≥5 to <16 years**<br>OR (95%CI)	**Adults**<br>**≥16 to (<60 women, <65 men) years**<br>OR (95%CI)
**Age (years)**	0.99 (0.98–0.99)	0.99 (0.84–1.17)	0.99 (0.98–1.01)
**Sex**			
Female	reference	reference	reference
Male	0.81 (0.63–1.04)	1.05 (0.43–2.52)	0.88 (0.62–1.24)
**Ethnicity**			
White	reference	reference	reference
Non-White	1.33 (0.70–2.61)	2.19 (0.44–16.49)	1.94 (0.68–6.98)
**NS-SEC**			
Managerial/professional	reference	reference	reference
Intermediate	0.86 (0.63–1.18)	1.19 (0.36–4.38)	1.01 (0.66–1.56)
Routine/manual	1.10 (0.80–1.50)	4.20 (0.99–29.21)	1.24 (0.81–1.90)
**Symptom severity**			
Mild	reference	reference	reference
Moderate	3.41 (2.56–4.56)	4.19 (1.62–11.79)	3.73 (2.56–5.49)
Severe	5.64 (4.15–7.72)	8.85 (2.56–41.76)	5.04 (3.30–7.81)
Number	1250	127	695

CI = confidence interval; IID = infectious intestinal disease; NS-SEC = National Statistics Socioeconomic Classification; OR = odds ratio

Sensitivity Analysis using Multiple Imputation

Multiple imputation by chained equations was used to estimate missing data values within the symptom severity, sickness absence and NS-SEC variables. Variables predictive of these three variables, variables that were predictive of data being missing within the three variables, and all of the variables used in the final regression models were included in the imputation model. In total, 40 imputed datasets were generated. Multivariate analyses using the most parsimonious models for the IID symptom severity and sickness absence outcomes identified in the main analysis were repeated using the imputed datasets.

Multivariate model for severe IID symptoms, versus mild or moderate symptoms combined for cases ≥5 years of age (IID2 study 2008–9)

	**Symptom severity**<br>OR (95%CI)
**Age group** (years)	
5–14	reference
15–24	2.35 (1.46–3.77)
25–44	1.08 (0.78–1.48)
45–64	0.72 (0.53–0.98)
65+	0.65 (0.47–0.88)
**Sex**	
Female	reference
Male	0.90 (0.75–1.07)
**Ethnicity**	
White	reference
Non-White	1.26 (0.80–1.97)
**NS-SEC**	
Managerial/professional	reference
Intermediate	1.26 (1.00–1.59)
Routine/manual	1.80 (1.43–2.27)
Number	1915

CI = confidence interval; IID = infectious intestinal disease; NS-SEC = National Statistics Socioeconomic Classification; OR = odds ratio

Multivariate models for sickness absence due to IID for cases of school/working age (IID2 study 2008–9)

	**Model 1**<br>OR (95%CI)	**Model 1 + Severity**<br>OR (95%CI)
**Age (years)**	0.98 (0.98–0.99)	0.99 (0.98–1.00)
**Sex**		
Female	reference	reference
Male	0.99 (0.78–1.25)	1.00 (0.77–1.29)
**Ethnicity**		
White	reference	reference
Non-White	1.78 (0.94–3.37)	1.63 (0.84–3.17)
**NS-SEC**		
Managerial/professional	reference	reference
Intermediate	1.04 (0.76–1.42)	0.96 (0.69–1.34)
Routine/manual	1.38 (1.00–1.89)	1.10 (0.79–1.54)
**Symptom severity**		
Mild		reference
Moderate		3.25 (2.41–4.37)
Severe		5.01 (3.62–6.93)
Number	1270	1270

CI = confidence interval; IID = infectious intestinal disease; NS-SEC = National Statistics Socioeconomic Classification; OR = odds ratio

Sickness Absence Duration amongst Absentee IID Cases

The relationship between NS-SEC and absence duration amongst absentee IID cases of school/working age was investigated using negative binomial regression. The incident rate ratios and 95% confidence intervals for absence duration in days, for three nested models are shown below. The addition of NS-SEC to the baseline model improved the model fit when comparing the likelihoods of the models (Likelihood ratio χ^2 7.36; P = 0.025). Those in routine/manual compared to managerial/professional occupations were more likely to be absent for longer due to IID (IRR 1.2, 95%CI; 1.04–1.40). However, when IID symptom severity was added to this model the association between NS-SEC and absence duration was attenuated.

Multivariate models for absence duration for absentee IID cases of school/working age (IID2 study 2008–9)

	**Baseline model**	**Baseline + NS-SEC**	**Baseline + NS-SEC + Severity**
	IRR (95%CI)	IRR (95%CI)	IRR (95%CI)
**Age (years)**	1.00 (1.00–1.01)	1.00 (1.00–1.01)	1.00 (1.00–1.01)
**Sex**			
Female	reference	reference	reference
Male	1.09 (0.96–1.23)	1.07 (0.94–1.21)	1.10 (0.98–1.24)
**Ethnicity**			
White	reference	reference	reference
Non-White	1.50 (1.18–1.90)	1.47 (1.15–1.86)	1.39 (1.10–1.73)
**NS-SEC**			
Managerial/professional		reference	reference
Intermediate		1.17 (0.99–1.38)	1.11 (0.94–1.30)
Routine/manual		1.21 (1.04–1.40)	1.08 (0.94–1.25)
**Symptom severity**			
Mild			reference
Moderate			1.34 (1.14–1.57)
Severe			2.02 (1.74–2.36)
Log-likelihood	-960.5	-956.8	-915.5
Deviance	431.0	428.9	401.8
AIC	1930.9	1927.5	1848.9
BIC	1952.0	1957.1	1886.9
Number	503	503	503

AIC = Akaike information criterion; BIC = Bayesian information criterion; CI = confidence interval; IID = infectious intestinal disease; IRR = incident rate ratio; NS-SEC = National Statistics Socioeconomic Classification
